# Fungal coinfection/superinfection in COVID-19 patients in a tertiary hospital in Mexico

**DOI:** 10.7705/biomedica.7251

**Published:** 2024-08-29

**Authors:** Eduardo García-Salazar, Sandra Benavidez-López, Alexandro Bonifaz, Emma Alejandra Hernández-Mendoza, Xóchitl Ramírez-Magaña, María del Rocío Reyes-Montes, Esperanza Duarte-Escalante, Gustavo Acosta-Altamirano, María Guadalupe Frías-De-León

**Affiliations:** 1 Hospital Regional de Alta Especialidad Ixtapaluca, IMSS-BIENESTAR, Ciudad de México, México IMSS-BIENESTAR México México; 2 Laboratorio de Micología, Servicio de Dermatología, Hospital General de México "Dr. Eduardo Liceaga", Ciudad de México, México Hospital General de México "Dr. Eduardo Liceaga" México México; 3 Departamento de Microbiología y Parasitología, Facultad de Medicina, Universidad Nacional Autónoma de México, Ciudad de México, México Universidad Nacional Autónoma de México Departamento de Microbiología y Parasitología Facultad de Medicina Universidad Nacional Autónoma de México México Mexico

**Keywords:** Mycoses, COVID-19, candidemia, aspergillosis, México, micosis, COVID-19, candidemia, aspergilosis, México

## Abstract

**Introduction.:**

Data on the prevalence of fungal coinfections/superinfections in patients with COVID-19 are limited.

**Objective.:**

To describe the prevalence of fungal coinfections/superinfections in patients with COVID-19, as well as risk factors and demographic, clinical, and microbiological characteristics.

**Material and methods.:**

We included patients with a confirmed COVID-19 diagnosis and a confirmed fungal infection hospitalized in the ICU from March 2020 to December 2021. We collected data on age, sex, comorbidities, hospital length of stay (days), laboratory (ferritin) and microbiological results, treatment for COVID-19, antifungal therapy, and outcomes obtained from the clinical records.

**Results.:**

Only 11 out of 740 patients met the inclusion criteria. The coinfection rate was 0.3% and the superinfection was 1.2%. The most affected population was male adults. The coinfections/superinfections diagnosed were candiduria and candidemia, caused by *Candida albicans, C. tropicalis, C. glabrata, C. lusitaniae,* and *Kluyveromyces marxianus (C. kefyr).* In addition, tracheobronchitis due to *Aspergillus fumigatus* was found. The most used antifungals were fluconazole and caspofungin. The lethality in patients with fungal coinfections was 50% and superinfections, 22%. The length of hospital stay was 11-65 days. Eight patients required mechanical ventilation and six received corticosteroids. The main comorbidity was diabetes mellitus (81.8%).

**Conclusions.:**

The rate of fungal coinfections/superinfections in COVID-19 patients was low, but the lethality found urges for routine fungal screening in patients with severe COVID-19 to timely detect fungal infections that may further compromise the patient's life.

## RESUMEN

**Introducción.:**

Los datos sobre la prevalencia de coinfecciones o sobreinfecciones fúngicas en pacientes con COVID-19 son limitados.

**Objetivo.:**

Describir la prevalencia de coinfecciones o sobreinfecciones fúngicas en pacientes con COVID-19, así como los factores de riesgo y las características demográficas, clínicas y microbiológicas.

**Material y métodos.:**

Se incluyeron pacientes con diagnóstico confirmado de COVID-19, hospitalizados en la unidad de cuidados intensivos y con infección fúngica confirmada entre marzo del 2020 y diciembre del 2021. Del expediente clínico se obtuvieron datos sobre edad, sexo, comorbilidades, días de estancia hospitalaria, resultados de laboratorio (ferritina) y microbiológicos, tratamiento contra COVID-19, terapia antifúngica y desenlace.

**Resultados.:**

Once de 740 pacientes cumplieron con los criterios de inclusión. La tasa de coinfección fue del 0,3 % y la de sobreinfección fue del 1,2 %. La población más afectada fue la de hombres adultos. Las coinfecciones o sobreinfecciones diagnosticadas fueron candiduria y candidemia, causadas por *Candida albicans, C. tropicalis, C. glabrata, C. lusitaniae* y *Kluyveromyces marxianus (C. kefyr).* Además, se encontró una traqueobronquitis por *Aspergillus fumigatus.* Los antifúngicos más administrados fueron fluconazol y caspofungina. La letalidad en pacientes con coinfecciones fue del 50 % y con sobreinfecciones fúngicas, del 22 %. El tiempo de estancia intrahospitalaria fue de 11 a 65 días. Ocho de los pacientes requirieron asistencia respiratoria mecánica y seis recibieron corticoides. La principal comorbilidad fue diabetes mellitus (81,8 %).

**Conclusiones.:**

La tasa de coinfecciones o sobreinfecciones por hongos en pacientes con COVID-19 fue baja, pero la letalidad de estas requiere, con urgencia, la realización de pruebas de rutina para detectar hongos en pacientes con COVID-19 grave para diagnosticar oportunamente infecciones fúngicas que puedan comprometer aún más la vida del paciente.

Similarly to what has occurred in other respiratory virus epidemics, during the coronavirus disease 2019 (COVID-19) pandemic -caused by severe acute respiratory syndrome coronavirus 2 (SARS-CoV-2)- various events arose that not only further complicated the therapeutic management of patients but also increased considerably the mortality rate, mainly in those who had a dysregulated immune system as they developed severe forms of the disease ^1^ -^5^.

The presence of coinfections caused by viruses, bacteria, or fungi at the time of the diagnosis of SARS-CoV-2 infection or the subsequent development of superinfections were among these events ^4^^-^^8^. Among fungal coinfections, the genus *Aspergillus* has stood out as the most frequently associated pathogen, while species of the genus *Candida* have been the most identified in superinfections, including the multidrug-resistant species *C. auris*^9^ -^11^. Other less frequently reported fungi among the population with COVID-19 are *Histoplasma capsulatum, Coccidioides* spp., *Cryptococcus* spp., *Pneumocystis jirovecii, Fusarium* spp., *Scedosporium* spp., *Trichosporon asahii, Mucor*spp., and *Rhizopus* spp.; the latter emerged causing a severe additional health problem, mainly in India ^12^ -^15^.

According to reports in different parts of the world, data on the prevalence of fungal coinfections/superinfections among COVID-19 patients are variable ^2^^,^^8^^,^^14^^,^^16^. Overall, the prevalence of coinfections was reported in the range of 4 to12.6%, and superinfections between 6 and 8% ^2^^,^^8^^,^^17^ -^19^. However, it is essential to consider that this wide range of prevalence may be due partly to the difficulty in differentiating COVID-19 from fungal infection based on clinical or radiological data alone. Therefore, it is likely that many fungal coinfections or superinfections have gone unnoticed because of these diagnosis limitations ^20^.

Unfortunately, data on the prevalence, etiology, and outcomes of fungal coinfection and superinfection cases in patients with COVID-19 are limited compared to data on bacterial or viral coinfections/superinfections ^8^. This situation prompts the need for continuously sharing information regarding the prevalence of coinfections and superinfections in hospitalized COVID-19 patients ^21^. Even though on May 5, 2023, the World Health Organization announced the end of the health emergency worldwide, after more than three years of announcing the pandemic, it does not mean that COVID-19 has stopped being a threat to global health. Thus, it is crucial to have a broad overview of coinfections and superinfections, especially fungal ones, to prevent, detect, and treat them promptly, improving the prognosis of affected people.

The objective of this study was to describe the prevalence of fungal coinfections/superinfections in patients hospitalized due to COVID-19, as well as risk factors, demographic, clinical, and microbiological characteristics in a "COVID hospital" in Mexico, during March 2020 to December 2021.

## Materials and methods

### Study design

An observational, retrospective, and cross-sectional study on patients hospitalized in the intensive care unit due to COVID-19, who presented associated fungal coinfections/superinfections. The study covered the period from March 17, 2020 -when the first COVID-19 patient was received- until December 31, 2021.

The study was approved by the hospital's research and research ethics committees (NR-15-2020). Due to the type of study, no informed consent was required from the patients. Patient-identifiable information was treated as confidential.

*Inclusion criteria.* Patients diagnosed with COVID-19 confirmed by molecular detection of the SARS-CoV-2 virus who were hospitalized from March 17, 2020, to December 31, 2021, and diagnosed with fungal infection (fungemia, urinary tract infection, among others).

*Exclusion criteria.* Patients with fungal isolation without clinical relevance *(Candida* spp. in bronchoalveolar lavage, sputum, and bronchial aspirates).

*Elimination criteria.* Patients with incomplete clinical records.

### Definitions

Coinfection was defined as a community-acquired infection diagnosed in the first 48 hours of hospital admission due to COVID-19. Superinfections were considered infections acquired 48 hours after hospital admission.

### Data collection

We reviewed the hospital's clinical laboratory information system records to identify intensive care unit patients with positive mycological results reported during the study period. *Candida* species were identified using the automated VITEK^®^ 2 (BioMérieux, Marcy l'Etoile, France) ^22^.

For this purpose, a suspension was prepared with pure subcultures of the yeast isolates in 0.45% NaCl solution, and the turbidity was adjusted to a 2.0 McFarland standard. With this suspension, the VITEK^®^ 2 system automatically filled, sealed, and incubated the individual test cards. The cards were incubated at 35.5 °C for 18 hours, and optical density readings were taken automatically every 15 minutes. From these readings, an identification profile was established and compared with the database, generating the final yeast identification. Final identifications listed as "excellent," "very good," "good," "acceptable," or "low discrimination" were considered correct.

The *Aspergillus* isolate was identified based on its macro- and micro-morphological characteristics ^23^.

After the patients were selected, their electronic medical records were reviewed through the Saludness system ^24^ to obtain the following information: demographic data (sex, age), comorbidities, hospital length of stay, treatment for COVID-19, risk factors for fungal infection, laboratory (ferritin) and microbiological results (blood, urine, and respiratory sample cultures), antifungal therapy and outcomes (mortality).

### Data analysis

Continuous data were presented as mean ± standard deviation (SD), while categorical data were given as absolute numbers and percentages.

## Results

During the study period, 880 COVID-19 patients were admitted to the hospital's intensive care unit, 740 had a positive SARS-CoV-2 PCR test. Isolation of fungal agents of the genus *Candida* or *Aspergillus* was reported in 26 patients. However, only 11 patients met the study inclusion criteria. The rate of fungal infection in COVID-19 patients was 1.5% (11/740).

Analyzing the data by infection subtype, we found that 9/740 (1.2%) of the included patients were diagnosed with fungal superinfections. Fungal coinfections were recorded in 2/740 (0.3%) patients only.

The time between the COVID-19 diagnosis and the detection of fungal infections was 2-60 days; 72.7% (8/11) of the population with fungal infection were males, and 27.3% (3/11) were females. The patients' age range was 22 to 72 years (50.4 ± 15.4).

The diagnosed superinfections were six cases of urinary tract infections, two of candidemia, and one of tracheobronchitis due to *Aspergillus fumigatus.* The coinfections were two cases of urinary tract infections. The causative agents of urinary tract infections were *Candida albicans* (n = 5), *C. tropicalis* (n = 1), and *C. glabrata* (n = 1), and one case had simultaneous isolation of *C. tropicalis* and *Kluyveromyces marxianus (C. kefyr).* Candidemias were caused by *C. tropicalis* (n = 1) and *C. lusitaniae* (n = 1).

Fungal superinfections were treated with fluconazole (n = 4), caspofungin (n = 4), and voriconazole (n = 1), while two patients did not receive antifungal treatment. One of these patients did not receive treatment because he died on the same day the mycosis was diagnosed, and the other patient requested to be transferred to another hospital to continue treatment. The case fatality rate among the study population was 27.3% (3/11). Looking at the infection subtype, we observed that the lethality in patients with fungal coinfections was 50% (1/2), while in patients who presented fungal superinfections it was 22% (2/9).

The length of hospital stay was 11 to 65 days (29.3 ± 17.5). Eight patients received mechanical ventilation for 21-34 days. It is worth mentioning that one patient required mechanical ventilation on two occasions (table 1).

**Table 1:** Characteristics of hospitalized COVID-19 patients who presented fungal coinfection/superinfection

Patient	Sex	Age (years)	COVID-19 treatment	Comorbidities	Hospital stay (days)	Predisposing factors	Ferritin (ng/ml)	Type of infection (superinfection/ coinfection)	Type of mycosis (etiology)	Antifungal treatment	Outcome (deceased/ cured)
1	Male	43	Azithromycin, piperacillin/ Tazobactam, linezolid, paracetamol	Ischemic CVD, cerebral edema, chronic ischemic heart disease, acute coronary syndrome, metabolic syndrome, obstructive sleep apnea, non-purulent moderate soft tissue infection, peripheral arterial insufficiency, acute kidney injury, DM2, morbid obesity	11	ICU	996.9*	Coinfection	Candiduria (*C. albicans*)	Did not receive	Deceased
2	Male	58	Methylprednisolone, cefepime, paracetamol	Alcoholism	56	ICU, mechanical ventilation, use of broad- spectrum antibiotics	936.4*	Coinfection	Candiduria (*C. albicans*)	Voriconazole	Cured
3	Male	51	Methylprednisolone, hydroxychloroquine sulfate, zithromycin, ceftriaxone	No comorbidities	23	Prolonged ICU, mechanical ventilation	673.9*	Superinfection	Candiduria (*C. albicans*)	Fluconazole	Deceased
4	Female	26	Dexamethasone, azithromycin, vancomycin	Myeloid leukemia	16	ICU	745.4*	Superinfection	Candiduria (*C. tropicalis* and *C. kefyr*)	Fluconazole	Cured
5	Male	44	Tocilizumab, nitric oxide, azithromycin, ceftriaxone	No comorbidities	15	ICU, mechanical ventilation	523.1*	Superinfection	Candiduria (*C. albican*s)	Fluconazole	Cured (the patient requested discharge while in serious condition)
6	Female	22	Dexamethasone, chloropyramine, paracetamol	Gestational DM2, acute lymphoblastic leukemia, obesity, post-hysterectomy pathological puerperium, obstetric hemorrhage, ischemic CVD, DM2, axillary DVT, moderate depression	21	ICU	900.9*	Superinfection	Candidemia (*C. tropicalis*)	Did not receive	Cured
7	Male	61	Paracetamol	DM2, SAH, stage 1 chronic kidney disease	24	ICU, mechanical ventilation	1622*	Superinfection	Candiduria (*C. albicans*)	Fluconazole	Deceased
8	Male	72	Levofloxacin, azithromycin	Mixed dyslipidemia, acute kidney injury - KDIGO I	40	ICU, mechanical ventilation	456.4*	Superinfection	Candidemia (*C. lusitaniae*)	Caspofungin	Cured
9	Female	58	Dexamethasone, paracetamol, meropenem	DM2, SAH, broad- spectrum antibiotics(vancomycin, amikacin, meropenem)	19	ICU, mechanical ventilation	528.3*	Superinfection	Candiduria (*C. glabrata*)	Caspofungin	Cured
10	Male	61	Dexamethasone, amikacin, vancomycin	DM2, vitamin D deficiency.	33	Prolonged ICU, required mechanical ventilation twice	696.4*	Superinfection	Candiduria (*C. tropicalis*)	Caspofungin	Cured
11	Male	59	Vancomycin, gentamicin	Tracheostomy, DM2, acute kidney disease, acute bile reflux gastropathy, SAH	65	ICU, mechanical ventilation	1159.3*	Superinfection	Tracheobronchitis (*A. fumigatus*)	Caspofungin	Cured (without sequelae)

ICU: Intensive care unit; CVD: Cardiovascular disease; DM2: Diabetes mellitus, type 2; DVT: Deep vein thrombosis; SAH: Systemic arterial hypertension; KDIGO: Kidney disease improving global outcomes

* Values outside the normal range

Of the 11 patients included in this study, 81.8% (9/11) presented comorbidities, the most frequent being type 2 diabetes mellitus (n = 6), followed by systemic arterial hypertension (n = 3), chronic kidney disease (n = 3), obesity (n = 2), dyslipidemia (n = 1), acute myeloid leukemia (n = 1), acute lymphoblastic leukemia (n = 1) and alcoholism (n = 1).

Regarding the treatment for COVID-19, six patients (54.5%) received corticosteroids (dexamethasone and methylprednisolone), and five of these patients received combined treatment with broad-spectrum antibiotics. Four patients (36.4%) received antibiotic therapy, with azithromycin being the most used, followed by ceftriaxone (n = 3), vancomycin (n = 3), cefepime (n = 2), amikacin (n = 2), levofloxacin (n = 1), meropenem (n = 1), and piperacillin/ tazobactam (n = 1). One patient (9.1%) was treated only with paracetamol.

## Discussion

In Mexico, the COVID-19 pandemic affected more than 7,614,771 people, killing 334,107 ^25^. This high lethality was mainly associated with diseases such as type 2 diabetes mellitus, systemic arterial hypertension, and cardiovascular diseases. However, it is essential to mention that fungal/bacterial/viral coinfections/superinfections probably contributed to this lethality too, as documented in other epidemics/pandemics caused by respiratory viruses, such as the ones causing SARS (Severe Acute Respiratory Syndrome), MERS (Middle East Respiratory Syndrome), influenza A (H_1_N_1_), parainfluenza, as well as rhinovirus, adenovirus, respiratory syncytial virus, and cytomegalovirus ^26^ -^30^.

The prevalence of secondary infections in each epidemic/pandemic has varied. For example, 25 to 30% of the SARS survivor population was estimated to develop a coinfection/superinfection by another microorganism ^31^. Unfortunately, COVID-19-associated infections have not been sufficiently explored, let alone secondary fungal infections ^32^. Therefore, in this study, we report the prevalence of fungal coinfections/superinfections in patients hospitalized due to COVID-19 in a hospital center dedicated to providing exclusive care for this condition during the pandemic. Our results yielded a rate of fungal coinfections/superinfections of 1.5%, showing that superinfections (1.2%) were more frequent than coinfections (0.3%). The previous finding coincides with that reported by Musuuza *et al.*^8^, who determined a frequency of 8% for superinfections and 4% for coinfections.

However, the rate of fungal coinfections/superinfections (1.5%) we reported was lower than those observed in other studies, which ranged from 4 to 12% ^1^^,^^2^^,^^8^^,^^17^ -^19^. This discrepancy is probably because those rates were calculated in systematic review studies and meta-analyses compiling all data reported on this topic from the end of 2019 to mid-2021 -the most complicated period of the COVID-19 pandemic- and, therefore, included many patients from different parts of the world ^1^^,^^2^^,^^8^^,^^17^ -^19^. However, despite the small number of patients included in our study, the data is consistent with those obtained by García-Vidal *et al.*^33^, who reported a low fungal infection rate (0.7%) in a Spanish population of 989 patients hospitalized for COVID-19.

In this way, some of the factors that may be involved in the low rate of fungal infection found were: 1) underdiagnosis, since fungal screening was not routinely performed in patients; and 2) limited diagnostic methods for mycoses, as only cultures were used for isolating pathogens, even though this is not the most sensitive diagnostic method ^34^. Unfortunately, some mycoses might have gone unnoticed as molecular methods were not applied.

This work data also showed that the most affected population with fungal coinfections/superinfections associated with COVID-19 were male adults in the intensive care unit with a median age of 58.5 years, which coincided with reports in other countries ^14^^,^^35^ -^37^.

In addition, following other reports, urinary tract and bloodstream infections caused by the genus *Candida* were the predominant infections in the studied population related to urinary catheters, prolonged hospitalization time, and broad-spectrum antibiotic treatments ^33^.

On the other hand, tracheobronchitis, characterized by plaques in the large airways (trachea and bronchi), was the only manifestation of invasive aspergillosis that we found in a patient who required mechanical ventilation for a prolonged period, which possibly predisposed to mycosis ^33^. Tracheobronchitis has been reported with a low frequency in 10-20% of critically ill COVID-19 patients ^38^^,^^39^. In addition, it has been speculated that the development of tracheobronchitis is most likely due to epithelial erosion caused by viral replication. This condition is a predisposing factor since the damaged epithelium may be a pathway that facilitates the entry, colonization, and proliferation of *Aspergillus* spp. in the respiratory tract ^40^.

Likewise, the primary etiological agent identified in candidiasis was *C. albicans,* while in aspergillosis it was *A. fumigatus.* Both pathogens have been the most reported in COVID-19-associated mycoses ^14^^,^^17^^,^^19^, although *Mucor* spp. and *Rhizopus* spp. are also frequently associated pathogens ^14^. In addition, the most used antifungal treatment for candidiasis was fluconazole and for aspergillosis it was caspofungin, contrary to the management with voriconazole that Sayedjavadi *et al.*^14^ reported while treating 181 COVID-19 patients coinfected mainly with *Aspergillus* spp., *Candida* spp., *Mucor* spp., and *Rhizopus* spp.

On the other hand, the 11 patients included in this study presented several conditions, not mutually exclusive, acting as predisposing factors to fungal infection or any other type of infection. Among these factors, we found in seven patients the inhibition of the immune system associated with corticosteroid treatment (methylprednisolone, dexamethasone) for COVID-19 and broad-spectrum antibiotics (azithromycin, ceftriaxone, vancomycin, cefepime, amikacin, levofloxacin, meropenem, and piperacillin-tazobactam), mechanical ventilation indicated for an extended period (21-34 days), prolonged hospital stay in the ICU (mean = 26 days), and elevated ferritin levels (456.4-1,622 ng/ml) ^36^^,^^41^^,^^42^.

In addition to these factors, comorbidities -particularly type 2 diabetes mellitus, systemic arterial hypertension, and chronic renal failure- possibly influenced severity and lethality in these patients. Likewise, this study evidenced a lethality of 50% in patients with fungal coinfections, like the results of Sayedjavadi *et al.*^14^, who determined a case fatality rate of 54.6% in a systematic review study of cases of COVID-19 patients with fungal coinfection reported on January 1, 2020, to November 30, 2021. In our study, the case fatality rate determined in COVID-19 patients with fungal superinfections was 22.2%. In this way, several authors have reported that coinfection and fungal superinfection negatively influence the progression and prognosis of the SARS-CoV-2 disease, particularly in critically ill patients ^8^^,^^9^^,^^14^.

It is crucial to mention that the interpretation of the COVID-19 impact on the development of fungal coinfections/superinfections must be cautious, as it involves different elements, such as the immune host defenses, the virulence of co-infecting pathogens, and risk factors ^43^. For example, in this study, *Candida* spp. coinfections/superinfections (candidemia and candiduria) were associated with frequent predisposing factors, for example, aspects that make any intensive care unit patient vulnerable, such as the use of broad-spectrum antimicrobials, mechanical pulmonary ventilation, and prolonged hospital stay ^44^.

One of the processes worth highlighting is the colonization of *Candida* spp. in various mucous membranes, which can lead to severe infection, especially candidemia. Therefore, COVID-19 apparently does not contribute to increased susceptibility to candidiasis ^43^. During the pandemic, the incidence of candidiasis increased ten times, particularly candidemias caused by *C. albicans,* but that increase was observed in COVID-19 patients and patients with other morbidities ^45^. Likewise, the mortality rate showed no statistically significant difference between the populations of COVID-19 patients and subjects without coronavirus infection ^46^. Therefore, it is complex to determine whether SARS-CoV-2 infection leads to secondary *Candida* infection ^47^.

Nonetheless, evidence suggests that severe viral infections, such as influenza A, can lead to concomitant *Aspergillus* spp. infections with a high mortality rate ^48^. A likely explanation is that phagolysosome maturation in neutrophils and monocytes is altered after viral infection, limiting the ability of these cells to eliminate *Aspergillus* spp. ^49^, in such a way that the fungus can proliferate easily causing infections in two forms: saprophytic pulmonary (aspergillomas) and invasive pulmonary.

Some studies have shown that influenza can not only affect local phagocytosis of alveolar macrophages but can also limit the functionality of natural killer cells, cause cytokine imbalance, affect the Th_1_/Th_2_ response, and generate lymphopenia, leading to increased patient vulnerability to secondary infections ^48^^,^^50^^,^^51^. In addition to impaired local immunity, the influenza virus can alter the respiratory epithelium, cause barrier function loss and decreased cilia movement, which may promote viral spread to the deeper lung parenchyma and create a favorable environment for the establishment of *Aspergillus* spp. and other pathogens ^43^^,^^48^.

However, the mechanism of intrinsic immune dysregulation caused by the influenza virus and SARS-CoV-2 is unknown. Recently, some pathophysiology-related hypotheses have been laid out that may explain the development of COVID-19-associated aspergillosis.

The first hypothesis is linked to the dysfunctional response of type I and III interferons (IFN) in severe COVID-19 patients, having a fundamental role against *Aspergillus* spp. because it promotes the response of type 1 T helper cells against the fungus and drives the production of type III IFN, causing neutrophils to act against *A. fumigatus.*

The second hypothesis is related to IFN dysregulation, along with cell depletion of alveolar macrophages, as they are the first cells to recognize inhaled conidia, thus being another possible cause of invasion by *Aspergillus* spp. ^52^ -^54^.

It is possible that the different variants of the SARS-CoV-2 coronavirus influence, to a greater or lesser extent, the incidence of secondary aspergillosis, similar to influenza A and B viruses ^48^. However, this is something that has yet to be studied.

This study has some limitations. At the time of hospital admission, patients were not screened for fungal pathogens, so possibly many coinfections were not detected, particularly in patients who died shortly after being admitted to the hospital. In addition, it is a retrospective study in which infections are reported based solely on mycological isolation documentation. The latter is disadvantageous as it is not always possible to isolate pathogens. Unfortunately, molecular tests were not performed to search and identify fungi in the study population, so fungal infections were likely underdiagnosed. However, our results support that patients with severe SARS-CoV-2 infection need to be routinely screened for both fungal and other microorganism infections promptly ^55^^,^^56^. This measure will be helpful for decision-making during the therapeutic management of patients and for achieving a better prognosis. Likewise, the use of empirical antifungal therapy at the early stage of the SARS-CoV-2 infection may be a wise choice, as some of the secondary infections are not linked to the respiratory system but are to the urinary tract or bloodstream ^2^^,^^18^, as shown in our findings.

In conclusion, the rate of fungal coinfections/superinfections in COVID-19 patients was low, with adult males being the most affected population. Urinary tract and bloodstream infections were the primary clinical forms caused by *C. albicans, C. tropicalis, C. glabrata, K. marxianus (C. kefyr),* and *C. lusitaniae.* Meanwhile, we only found one case of tracheobronchitis by *A. fumigatus.* Prolonged hospital stays, extended mechanical ventilation, corticosteroids, and broad-spectrum antibiotics, as well as diabetes mellitus, were the main risk factors for developing fungal infections. However, the interpretation of the COVID-19 impact on the development of fungal coinfections/superinfections must be cautious. Based on these findings, we recommend routine screening to identify fungi in patients with severe SARS-CoV-2 to timely detect fungal infections that may further compromise the patient's life.
